# Meta Mesh Ontology for Cross-Domain Mental Health Knowledge Integration: Development and Evaluation Study

**DOI:** 10.2196/96311

**Published:** 2026-10-23

**Authors:** Rogério Blitz, Maike Richter, André Kerber, Ramona Leenings, Sylvia Thun, Nils Opel

**Affiliations:** 1 Department of Psychiatry and Neurosciences, Charité – Universitätsmedizin Berlin, Campus Benjamin Franklin, Hindenburgdamm 30, Berlin, 12203, Germany, 49 30 450 517 522 ext 522; 2 German Center for Mental Health (DZPG), partner site Berlin/Potsdam, Berlin, Germany; 3 Division of Clinical Psychological Intervention, Freie Universität Berlin, Berlin, Germany; 4 Department of Neurology, Johannes Kepler University Linz, Linz, Austria; 5 Core Unit Digital Medicine and Interoperability, Berlin Institute of Health at Charité - Universitätsmedizin Berlin, Berlin, Germany

**Keywords:** mental disorders, precision medicine, biomedical ontologies, health information interoperability, data integration, knowledge bases, digital health, medical informatics, telemedicine, meta mesh ontology, semantic interoperability, ontology-based psychiatry

## Abstract

**Background:**

Psychiatric diagnostic practice faces persistent challenges in adequately capturing the complexity, heterogeneity, and dynamic nature of mental disorders. Conventional categorical classification systems such as the *Diagnostic and Statistical Manual of Mental Disorders, Fifth Edition (DSM-5)* and the *International Classification of Diseases, 11th Revision (ICD-11)* rely on rigid symptom-based criteria that often fail to represent multifactorial etiologies, dimensional symptom profiles, and high rates of comorbidity. Although dimensional models, domain-specific ontologies, and alternative taxonomies have been developed to address these limitations, they are typically applied in isolation, resulting in fragmented knowledge representations and limited interoperability. This lack of semantic integration hampers data harmonization and cross-domain analysis.

**Objective:**

The objective of this work is to develop and present the Meta Mesh Ontology (MMO) and its Core Domain Framework (CDF) as an integrative approach for mapping heterogeneous psychiatric knowledge bases and evaluating ontology structure, logical consistency, and specified retrieval and inference behavior.

**Methods:**

We designed a modular ontology architecture grounded in the Basic Formal Ontology to ensure structural and semantic consistency across biological, psychological, and social domains. Existing psychiatric frameworks and ontologies—including dimensional models, diagnostic taxonomies, and socioenvironmental knowledge systems—were systematically analyzed and represented through domain modules using a documented alignment and mapping procedure. The MMO was implemented using formal semantic web standards, including the Web Ontology Language and the Resource Description Framework (RDF), and aligned with established biomedical ontologies and informatics standards to support explicit mappings and modular extensions across domains. Evaluation used 2 MMO and CDF metric sets and 10 module metric sets to quantify ontology size, semantic alignments, external integration, and dataset readiness. We also assessed Web Ontology Language 2 Description Logic (OWL 2 DL) conformance, logical consistency, and 11 competency questions (including 1 entailment test) and 4 negative controls, in an outpatient scenario with 50 variables.

**Results:**

The MMO provides a shared semantic framework for representing links among heterogeneous psychiatric knowledge sources. Its modular design allows domain-specific subontologies to be incorporated under a common architecture. MMO and CDF comprised 2238 triples, 208 classes, 28 properties, 3 individuals, and 103 alignments (100 external) across 5 frameworks. The 10 modules added 967 triples and 171 alignments (116 external), with 21 integrations and complete label coverage (54/54 resources). The ontology resources conformed to OWL 2 DL; HermiT (Information Systems Group) detected no logical inconsistency, and all 11 competency questions and 4 negative controls produced the expected results. All 50 variables were linked to at least 1 CDF anchor, and 47 had an explicit Simple Knowledge Organization System (SKOS) mapping.

**Conclusions:**

The MMO provides a modular foundation for representing links among diverse psychiatric knowledge systems and data modalities. The current evaluation characterizes its structure, mappings, and behavior in the specified tests. Further research should assess mapping validity, governed clinical data, operational performance, and clinical utility.

## Introduction

### Background

Developments in digital psychiatry highlight the need for structured, interoperable, and context-sensitive data frameworks capable of capturing the complex interplay of biological, psychological, and social factors in mental disorders. Addressing this gap requires approaches that enable integrated, semantically coherent, and ethically responsible representations of mental health information.

### Challenges in Psychiatric Diagnostics

Psychiatric diagnostics face the challenging task of comprehensively capturing the multifaceted and often intertwined factors of mental disorders. This involves not only the precise identification and classification of symptoms but also the consideration of biological, social, and psychological influences that shape the individual clinical picture [1-5]. Despite their complexity, current diagnostic approaches remain incomplete, lacking consistent methods to account for socioeconomic parameters that significantly affect patients’ living conditions and overall well-being. In the context of personalized psychiatry, the goal is to develop individualized treatment approaches based on precise and differentiated diagnostics that are optimally tailored to the specific needs and living conditions of each patient. This aims to maximize therapeutic efficacy and sustainably improve the prognosis for affected individuals. Traditional classification systems, such as the *Diagnostic and Statistical Manual of Mental Disorders, Fifth Edition (DSM-5*) [6,7] and the *International Classification of Diseases, 11th Revision (ICD-11)* [8-10], which focus on symptomatic criteria, often reach their limitations in this regard. Their structure is designed to capture mental disorders within rigid, predefined categories, which frequently fail to adequately reflect the diversity and dynamics of these conditions [11]. This limitation becomes particularly evident in patients with complex symptom constellations and comorbidities, where diagnostic precision suffers, making tailored therapeutic approaches more difficult. Furthermore, the high variability of symptoms among different patients presents a significant challenge. Even with identical diagnoses, mental disorders can manifest in highly diverse ways and be subjectively experienced differently, complicating standardized diagnostics; for example, borderline personality disorder can encompass up to 256 possible symptom combinations. This diversity is influenced by cultural, social, and biological factors that are often not sufficiently considered in the diagnostic process. Incorporating individual life circumstances and patients’ biographical histories into both the diagnostic process and the formulation process is therefore essential for ensuring a holistic assessment of both mental and physical health aspects [12].

### Integration of Alternative Knowledge Representation Systems

Alternative knowledge representation systems, such as the Hierarchical Taxonomy of Psychopathology (HiTOP) [13,14] and the Research Domain Criteria (RDoC) framework [15], have been developed to capture the multicausal and interactive nature of mental disorders. These systems offer a more dynamic perspective that extends beyond traditional, rigid diagnostic typologies, considering the wide range of symptoms and their interactions [14]. However, a key limitation is that these systems are often applied in isolation and remain predominantly symptom-focused, thus overlooking crucial contextual information, including risk factors and social determinants of mental health. The lack of standardized and interoperable codifications significantly hampers the use of these alternative knowledge representation systems in both clinical practice and research [16,17]. Unified data formats and classification schemes are required to effectively integrate and consolidate data, enabling coherent data harmonization. The absence of such uniform standards results in the loss of valuable information, either by not being systematically captured or shared across different institutions and studies. A comprehensive networking and integration of heterogeneous data systems is currently only partially possible, which severely hinders the development of truly personalized and dynamic diagnostics because disconnected and nonharmonized data sources do not allow the correlation of biological, psychological, and social factors, thereby preventing a holistic understanding of individual patient trajectories. These challenges are not only technical but also organizational, as many clinics and research institutions use different systems and approaches. This lack of integration not only limits diagnostic precision but also the development of individualized treatment strategies and the understanding of underlying biopsychosocial patterns. Related ontology-based approaches have also been applied to noncommunicable and vector-borne disease modeling using Semantic Web Rule Language (SWRL)-based semantic rules [18,19], illustrating a broader trend toward semantic integration in clinical knowledge representation outside psychiatry. This paper addresses these challenges by introducing the Meta Mesh Ontology (MMO) [20,21], a new integrative framework designed to harmonize heterogeneous data sources and enable the semantic interconnection of clinical, biological, environmental, and social information. The MMO provides the methodological foundation for a unified, scalable, and ethically grounded representation of psychiatric knowledge. Building on this architecture, future studies may examine the use of MMO-structured data in AI applications in psychiatry. Such applications would require separate development and evaluation before conclusions could be drawn about explainability, clinical applicability, diagnosis, prognosis, or personalized treatment planning.

In this study, we define the layered MMO and Core Domain Framework (CDF) architecture, provide its Resource Description Framework (RDF) and Web Ontology Language (OWL) implementation and 10 domain modules, document a mapping workflow, and evaluate ontology structure, alignment patterns, logical consistency, and competency questions defined before testing in a synthetic mental health scenario. We do not assess clinical deployment, patient outcomes, machine-learning models, operational scalability, or interoperability between deployed information systems. These remain topics for future evaluation.

## Methods

### Terminology and Architectural Scope

Throughout this study, we use ontology to denote a formal, machine-readable specification of classes, relations, axioms, and annotations, and knowledge base to denote an ontology combined with instance-level assertions. Knowledge representation, by contrast, is the broader family of schemes used to encode domain knowledge, among them ontologies, terminologies, taxonomies, and classification systems. Within this architecture, the MMO core defines entities and relations shared across the domain modules at the meta level, while the CDF supplies anchors that connect them across domains. Here, the meta level denotes the domain-independent layer of MMO classes and relations reused by the domain modules. An anchor is an identified CDF entity to which a module class is linked to express its position within the cross-domain architecture. A domain module is, in turn, a separately versioned, MMO-aligned component linking selected external concepts to the CDF; external systems are not necessarily imported in full, and for each module we distinguish references, asserted mappings, transformations, and imports. In this context, versioning means that changes are recorded in identifiable releases with immutable tags and archived release identifiers, allowing the exact evaluated artifacts to be retrieved. MMO-aligned means that a module uses stable identifiers, applies the documented MMO and CDF relations, records mapping provenance, and passes the specified release checks. A mapping is understood as a named relation between identified source and target entities, accompanied by its own provenance. Semantic interoperability, as used here, refers specifically to machine-readable mappings and to successful cross-module retrieval within the released resources. Table 1 summarizes these terms and their roles within the architecture.

**Table 1.** Key terminology and architectural components of the MMO^a^.

Term	Definition in this study	Implemented artifact
MMO core	Domain-independent ontology entities and relations shared across the domain modules at the meta level.	MMO, version 1.0.1 [20]
CDF^b^	Identified cross-domain entities used as anchors between module classes and broad knowledge domains.	CDF, version 1.0.1 [21]
Domain module	Separately versioned, MMO-aligned ontology component that connects selected source concepts to the MMO core and CDF using documented relations and provenance.	Ten domain modules indexed in the MMO Modules Registry, version 1.0.0 [22]
External knowledge system	Referenced ontology, terminology, taxonomy, or classification system	Source URI^c^ and mapping predicate recorded in the relevant module [22]
Use-case knowledge base	Use-case instance assertions linked to MMO, CDF, module, and external entities	MMO use-cases, version 1.1.0 [23]

a MMO: Meta Mesh Ontology.

b CDF: Core Domain Framework.

c URI: Uniform Resource Identifier.

### Mathematical Foundation of the MMO

The mathematical framework underlying the MMO is formalized as an extended typed property-graph model. This is intended to represent hierarchical relations, context-dependent weights, and extensible metadata and permits the addition of domains and higher-order domain levels. The model is defined over a family of finite graph instances ${\left\{G_{r}\right\}}_{r\in N}$. Throughout the definitions below, the index *r* indicates that all indexed sets and functions belong to the same graph instance *G_r_* ; it is retained to distinguish properties of an individual finite instance from properties of the model family. No global constant N is imposed that bounds the number of nodes, edges, hierarchy levels, or admissible metadata keys in every graph instance. Extensibility therefore denotes the potential unboundedness of the model family, whereas each graph *G_r_* remains finite.

### Nodes and Weightings

In this model, the nodes represent typed entities, including data points and domains, and may be assigned context-dependent weights that reflect their role within the graph. For a graph instance indexed by *r* , let *V_r_* denote its finite set of nodes. We distinguish the disjoint subsets *P_r_* of data-point nodes and *D_r_* of domain nodes:


$$V_{r}\text{=}P_{r}\dot{\cup }D_{r}$$

The symbol $\dot{\cup }$ denotes a disjoint union of node types; it does not define a domain as the set-theoretic union of its subordinate nodes. The typing function


$$\tau_{V,\text{\textbackslash{} }r}:\text{\textbackslash{} }V_{r}\to T_{V}$$

assigns each node a type from the node-type set *T_V_* $T_{V}$. The data-point nodes may be enumerated as


$$P_{r}\text{=}\left\{\text{\textbackslash{} }p_{1}\text{\textbackslash{} },\text{\textbackslash{} }p_{2}\text{\textbackslash{} },\text{\textbackslash{} }\ldots \text{\textbackslash{} },\text{\textbackslash{} }p_{n_{r}}\text{\textbackslash{} }\right\}\text{\textbackslash{} }with\text{\textbackslash{} }n_{r}\in \text{\textbackslash{} }N$$

The finite cardinality *n_r_* depends on the graph instance and is not subject to a fixed global upper bound across the model family.

A node x ∈ *V_r_* may be associated with a context-dependent weight. For each weighting context *c ∈ C*, the partial function


$$\omega_{V,\text{\textbackslash{} }c,\text{\textbackslash{} }r}:\text{\textbackslash{} }V_{r}⇀R$$

assigns a real-valued weight when a weight is defined for *x*. The subscript *V* identifies this as a node-weighting function. The weighting function provides a formal mechanism for quantifying the relevance or affiliation of ontological entities within the property-graph model. It denotes the proportional assignment of a class or data point to one or multiple knowledge domains and serves both ontological and integrative purposes. On the ontological level, it expresses the degree to which a concept belongs to a given domain; on the integrative level, it quantifies the proportional affiliation of individual data points; on the data level, it describes the evidential strength or frequency within empirical datasets; and on the analytical level, it reflects the contextual importance within a modeling or classification task. The weighting contexts use the same formal codomain R but represent different constructs and must not be combined without an explicitly defined transformation or aggregation rule. Furthermore, each node x ∈ *V_r_* may carry an extensible set of metadata attributes.

If *K* denotes the set of metadata keys and *L* the set of literal values or resource identifiers, then


$$\mu_{V,r}:V_{r}\to P_{fin}(K\times L)$$

where *P_fin_* denotes the set of finite subsets. No global upper bound is imposed on the metadata keys that may be introduced across the model family, although the metadata instantiated for any node in an individual graph remains finite.

### Domains and Hierarchical Levels

A data point *p* ∈ *P_r_* may be associated with one or more domain nodes *d* ∈ *D_r_* . These associations are represented by edges whose source, target, and type functions are defined in the Edges and Relationships subsection. Each domain is itself a node in the graph and may have context-dependent weights and extensible metadata as defined above. Domains may in turn be connected to higher-order domains by edges assigned a hierarchical type, allowing the same hierarchical relation to be applied recursively across successive levels.

Let *L_r_* be the finite number of hierarchy levels instantiated in graph *G_r_* . A level-membership function


$$\lambda_{r}\text{\textbackslash{} }:\text{\textbackslash{} }D_{r}\text{\textbackslash{} }\to \text{\textbackslash{} }P_{fin}\left(\left\{1,\text{\textbackslash{} }\ldots ,\text{\textbackslash{} }L_{r}\right\}\right)\text{\textbackslash{} }\setminus \text{\textbackslash{} }\left\{\emptyset \right\}$$

assigns each domain node to 1 or more levels. Accordingly,


$$D_{r,l}\text{=}\left\{d\in D_{r}\right|l\in \lambda_{r}(d)\}$$

The sets *D_r,l_* need not be disjoint, because the same domain node may participate at more than 1 hierarchical level. Level membership is specified explicitly rather than derived from a unique path length. Because the model permits multiple hierarchical parents and permits a domain node to participate at more than one level, the hierarchical subgraph need not be a rooted tree and a unique distance from a single highest node need not exist. The function λ_*r*_ records this potentially multivalued level membership, whereas the hierarchical edges record the directed relations between domain nodes. Domains at level *l* are connected to domains at level *l*+1 through directed hierarchical edges. Let *^E^H,r* be the set of hierarchical edges in *G_r_*, and let *s_r_* and *t_r_* assign the source and target of an edge. The direct parents of a domain *d* are defined as


$$parent_{r}\left(d\right)\text{=}\left\{q\text{\textbackslash{} }\in \text{\textbackslash{} }D_{r,l\text{+}1}\text{\textbackslash{} }|\text{\textbackslash{} }\exists e\text{\textbackslash{} }\in \text{\textbackslash{} }E_{H,r}\text{\textbackslash{} }:\text{\textbackslash{} }s_{r}\left(e\right)\text{=}d\text{\textbackslash{} }\wedge \text{\textbackslash{} }t_{r}\left(e\right)\text{=}q\right\}$$

This definition permits a domain to have more than 1 higher-order parent while preserving each domain as an individual graph node. The terms domain and superdomain describe a node’s relative position in the hierarchy: a node may be a superdomain relative to a lower level and a subdomain relative to a higher level. If a particular application requires weights to be propagated across hierarchy levels, it may define a context-specific aggregation function *F_c_* such that


$$\omega_{V,c,r}\left(d\right)\text{=}F_{c}(\left\{\omega_{V,c,r}\left(q\right)\right|q\in {parent}_{r}(d)\})$$

The choice of *F_c_* is application dependent. The MMO does not prescribe an unqualified sum of all higher-level weights as a universal ontological rule.

### Edges and Relationships

For graph *G_r_ , E_r_*, is the finite set of directed edges. The functions


$$s_{r},t_{r}:E_{r}\to V_{r}$$

assign the source and target of each edge, and


$$\tau_{E,r}:E_{r}\to T_{E}$$

assigns an edge type. Edge types include hierarchical relations, ontology-structural relations, cross-system mappings, and instance-level assertions. This definition allows several differently typed edges between the same pair of nodes. For each context *c* ∈ *C*, optional edge weights are represented by


$$\omega_{E,c,r}\text{\textbackslash{} }:\text{\textbackslash{} }E_{r}\text{\textbackslash{} }⇀\text{\textbackslash{} }R$$

Edge metadata are represented by


$$\mu_{E,r}:E_{r}\to P_{fin}(K\times L)$$

### Recursive Hierarchy

The recursive character of the MMO lies in the repeated application of the same typed hierarchical relation across successive domain levels. Starting from *D*_*r,1*_, the levels instantiated in *G_r_* are obtained recursively through the parent relation up to the finite level *L_r_*. Let


$$I\text{=}\left\{G_{r\text{\textbackslash{} }}|\text{\textbackslash{} }r\text{\textbackslash{} }\in \text{\textbackslash{} }N\right\}$$

denote the family of admissible finite MMO graph instances. The MMO places no fixed global upper bound on the number of nodes, edges, hierarchy levels, or metadata keys permitted across this family. More precisely, for each of these quantities and every N\ ∈\ N, there exists an instance *G*_*r*_ ∈ *I* for which that quantity exceeds *N*.

### Overall Graph Structure

In summary, each graph instance of the MMO can be represented as a finite, directed, typed property graph with nodes and edges. Nodes may participate in hierarchical and nonhierarchical relations, and both nodes and edges may carry extensible metadata and context-dependent weights. The recursive application of hierarchical relations permits the introduction of additional domain levels, while the model family remains potentially unbounded as defined above. Formally, each MMO graph instance is represented as


$$G_{r}\text{=}\left(V_{r},\text{\textbackslash{} }E_{r},s_{r},t_{r},\tau_{V,r},\tau_{E,r},\lambda_{r},\mu_{V,r},\mu_{E,r},{\left\{\omega_{V,c,r}\right\}}_{c\text{\textbackslash{} }\in \text{\textbackslash{} }C},{\left\{\omega_{E,c,r}\right\}}_{c\text{\textbackslash{} }\in \text{\textbackslash{} }C}\right)$$

where:

1. *V*_*r*_ and *E*_*r*_ are the finite sets of nodes and directed edges, respectively;
2. *s*_*r*_ and *t*_*r*_ assign the source and target node of each edge;
3. ^*τ*^*V,r* and ^*τ*^*E,r* assign node and edge types;
4. *λ_r_* records the hierarchical-level membership of domain nodes;
5. *^μ^V,r* and *^μ^E,r* assign finite sets of metadata attributes to nodes and edges; and
6. {*^ω^V,c,r*} *c* ∈ *C, {^ω^E,c,r} c ∈ C* are the families of context-specific partial weighting functions for nodes and edges. A weighting function may remain undefined for an entity in a context in which no weight has been instantiated.

A domain node may participate at multiple hierarchical levels, and the edges between nodes form a network of relationships that can be analyzed across multiple dimensions. Because both nodes and edges may be associated with extensible metadata, each connection between entities is not only defined by its relation type and, where defined, its context-dependent weight but also by additional contextual information. This structure enables the MMO to represent mappings among heterogeneous knowledge representations and provides a formal basis for multidimensional analysis, semantic interoperability, and adaptation to complex knowledge systems. The extended property-graph model is a design-level abstraction of the MMO architecture rather than an alternative formal semantics for OWL. At the implementation level, RDF resources correspond to represented entities, RDF assertions correspond to directed typed relations, and annotations and provenance statements provide metadata. For example, the synthetic resource DP02_Dyssomnia is typed by a SNOMED module class, and explicit MMO predicates connect that class to a CDF anchor and external source identifiers; the complete path is shown in Section S7 in Multimedia Appendix 1. RDF and OWL additionally provide description-logic axioms and open-world entailment, which are evaluated separately by an OWL reasoner. The explicit level-membership and weighting functions extend the design abstraction beyond ordinary OWL class axioms.

### Module Alignment and Mapping Procedure

Module development followed a common procedure. We first defined each module’s scope and intended use and identified the relevant source ontology, terminology, classification system, or technical framework based on domain relevance, source authority, and the availability of identifiable concepts. From each source, we selected a bounded set of concepts sufficient to represent the use case and represented each concept as a module entity with a stable URI, source identifier, label, definition, and provenance annotation. Each entity was assigned to an MMO category and, where applicable, a CDF domain anchor. Mappings to source systems and other external resources were added where a semantically supportable correspondence was identified, with predicates selected according to the intended strength and direction of the relation. The 10 modules each contributed 5 exemplar concepts, yielding the 50 data points documented in Sections S4 and S5 in Multimedia Appendix 1. We used skos:exactMatch for concepts considered interchangeable within the stated mapping context, skos:closeMatch for highly similar but noninterchangeable concepts, and skos:relatedMatch for associative correspondences. The MMO-specific predicate mmo:isMappedTo records a cross-system link without asserting OWL equivalence, whereas mmo:isConstantRefinementOf links a more specific module entity to an MMO or CDF anchor. Source provenance was recorded through source identifiers and annotations, including dcterms:source and mmo:hasSource where applicable. Definitions, domain assignments, and mappings were curated iteratively by the multidisciplinary development team. Clinical specialists reviewed psychiatric meaning and domain relevance; ontology and medical-informatics specialists reviewed placement, provenance, and mapping predicates; and mathematics and computer-science specialists reviewed formal and technical consistency. Items requiring revision were discussed and revised until agreement was reached. The RDF and XML, Turtle, and OWL representations were subsequently assessed using the project-specific, version-controlled quality-control workflow shared across the MMO repositories; detailed configurations and outputs are available in the versioned releases [20-23].

### Storage and Accessibility

The MMO was implemented and published using standardized ontology formats, such as OWL [24] and RDF [25], which enable structured and formal descriptions of concepts, classes, attributes, relationships, and rules. In the RDF and OWL implementation, entities and typed relations are represented as RDF resources and predicates, with type information provided through RDF type assertions and OWL declarations. In the current implementation, mmo:isMappedTo, mmo:isConstantRefinementOf, and the Simple Knowledge Organization System (SKOS) mapping predicates record asserted relations between identified entities. Unless an OWL equivalence or subclass axiom is stated separately, these relations do not cause the connected entities to be inferred as equivalent to, or subclasses of, one another. The general weighting framework described above is an architectural provision for future analytical use; it is not currently instantiated with concrete weight values, metadata representations, or aggregation rules in the released RDF and OWL files (version 1.0.1). Persistent and versioned identifiers were established through recognized repositories and version-controlled archives [20-22], ensuring long-term reproducibility and traceability. Each ontology element, including classes, properties, and modules, is assigned a unique URI and a persistent URL [26,27], allowing consistent referencing across systems. The ontology is indexed via BioPortal [28] and the Ontology Lookup Service (OLS) [29], enabling structured access, semantic search, and cross-ontology linking. Source files, implementation documentation, and example datasets are maintained in the public GitHub repository [20], which also serves as the primary channel for feedback and version tracking [30]. The ontology is distributed under the Creative Commons Attribution 4.0 International (CC BY 4.0) license [31], supporting open reuse and adaptation. This infrastructure supports technical accessibility, version traceability, and reproducible reuse of the released resources.

### Quantitative Ontology Evaluation

We quantified ontology structure and asserted alignments independently of the functional evaluation. We performed a quantitative evaluation of the ontology framework using 2 complementary metric reports. The first report captured combined metrics for the MMO and the CDF. The second was an aggregate report across 10 domain modules spanning clinical, psychosocial, molecular, urban, and terminology domains (Adverse Childhood Experiences Ontology [ACESO], AWARE, Gene Ontology [GO], HiTOP, *International Classification of Diseases, Version 10 [ICD-10]*, International Organization for Standardization [ISO] 37120, Logical Observation Identifier Names and Codes [LOINC], Systematized Nomenclature of Medicine Clinical Terms [SNOMED CT], Social Determinants of Health Ontology [SOHO], and Unified Medical Language System [UMLS]). For the current study, dataset-readiness outputs were treated as ontology-side indicators reflecting concept-label coverage and schema annotations (eg, rdfs:label, rdfs:comment, and domain and range declarations), rather than as direct measures of empirical dataset interoperability. Integration counts were assessed at the module-report level, while external frameworks were recorded separately from aggregated module-level integration instances. The analysis included standard ontology size indicators (RDF triples, classes, properties, and individuals), as well as structural relations, semantic alignments, and integration signals within the framework. Semantic alignments were distinguished between internal project relations linking MMO modules to the CDF and external mappings to established reference frameworks. The evaluation further captured the number of external ontologies integrated into the framework. All quantitative metrics were computed programmatically using a Python (Python Software Foundation)-based analysis pipeline [32]. The pipeline parsed RDF and XML ontology files, extracted structural elements and semantic relations, and generated metric summaries. Additive metrics were aggregated across ontology components using merge and aggregation functions. Integrated external frameworks were consolidated through set-union operations to prevent double counting when the same external ontology appeared in multiple modules. Combined MMO and CDF metrics were calculated from the 2 ontology sources representing the core framework layers. Module-level metrics were computed independently for 10 MMO modules and subsequently aggregated to produce descriptive statistics across modules. This procedure enabled comparison of structural characteristics and semantic alignment patterns between modules. The evaluation also documented the semantic alignment predicates used in the framework. These included MMO-specific mapping relations (mmo:isConstantRefinementOf and mmo:isMappedTo) as well as standard semantic web alignment properties such as SKOS [33] exactMatch, closeMatch, and relatedMatch. External alignment targets comprised widely used biomedical and knowledge organization systems, including Open Biological and Biomedical Ontologies (OBO), SNOMED CT [34,35], *ICD-10* [36], LOINC [37,38], UMLS [39,40], and HiTOP [13], together with additional domain-specific or policy-related frameworks. All scripts used for metric extraction, aggregation, and report generation are publicly available in the project repositories on GitHub to support methodological transparency and reproducibility. All values were generated from MMO version 1.0.1, CDF version 1.0.1, and 10 domain-module releases at version 1.0.0 indexed in Modules Registry version 1.0.0 [20-22]; machine-readable outputs and reproduction scripts are available in the corresponding repositories.

### Ontology Verification and Competency-Question Validation

We distinguished descriptive characterization, verification, and validation following the conventional distinction between checking conformance to specified requirements and assessing whether an artifact supports its intended use [41]. The quantitative analyses described above characterize ontology structure and asserted alignments; they are not consistency tests. For verification, we evaluated MMO version 1.0.1, CDF version 1.0.1, and the 10 domain modules at version 1.0.0 using the project-specific, version-controlled workflow shared across the MMO repositories. The workflow parsed the RDF, XML, and Turtle serializations, assessed OWL 2 DL profile conformance, and applied HermiT reasoning through ROBOT version 1.9.6. Verification was performed separately for the MMO core, CDF, and each domain module. HermiT is an OWL 2 description-logic reasoner that evaluates the logical consequences of ontology axioms and can test ontology consistency under OWL 2 DL semantics [42]. A result of no detected inconsistency is limited to the released axioms and the applied reasoning procedure and does not establish the validity of every mapping. Validation comprised 11 predefined competency questions addressing cross-domain retrieval, mapping traceability, traversal from module entities to CDF anchors, provenance, integrity constraints, and 1 selected OWL entailment. SPARQL Protocol and RDF Query Language (SPARQL) queries were executed using RDFLib version 7.5.0, and the selected entailment was evaluated by Web Ontology Language 2 Rule Language (OWL-RL) materialization using OWL-RL version 7.1.4. Expected results and pass criteria were specified before execution. Four negative controls containing predefined violations were used to test whether the relevant validation procedures failed as expected. The 11 explicit questions, expected and observed results, and negative controls are reported in Section S6 in Multimedia Appendix 1; the worked mapping path and query are reported in Section S7. Complete executable queries, fixtures, software configurations, reports, and reproduction instructions are archived with the versioned evaluation release [23].

### Ethical Considerations

The mental health use case presented in this paper is a synthetic semantic modeling scenario constructed to demonstrate how heterogeneous clinical, social, environmental, and behavioral variables can be organized within the MMO and CDF architecture. No patient-level dataset, clinical record, participant observation, or identifiable information was generated or analyzed for this report, and the patient, encounter, and 50 listed data points are fictional exemplars rather than a reported clinical cohort. The study did not involve human participants or interaction with a clinical institution, production electronic health records (EHRs), or a live data platform. Accordingly, institutional review board approval and informed consent were not applicable.

## Results

### Introduction to the MMO

To address the outlined shortcomings, we introduce the MMO [20], a unifying framework designed to integrate heterogeneous knowledge bases within a coherent and interoperable structure. The MMO functions as a meta-ontology, acting as middleware between heterogeneous knowledge systems. In this capacity, it serves as an intermediary between various ontologies and knowledge representations, as illustrated in Figure 1.

Figure 1 presents the conceptual layers of the MMO architecture, progressing from heterogeneous raw knowledge sources and their formalized representations through the CDF and domain-module layer to the MMO middleware and its grounding in Basic Formal Ontology (BFO). In the released implementation, the architecture comprises the MMO core, the CDF, 10 separately versioned domain modules, and a use-case knowledge base [20-23]. The framework uses explicit mapping and refinement relations to connect selected entities while preserving the identity and provenance of the source representations. External ontologies and terminologies are not necessarily imported in full. New modules are incorporated as explicitly scoped, mapped, provenance-documented, versioned, and validated ontology artifacts.

**Figure 1.**
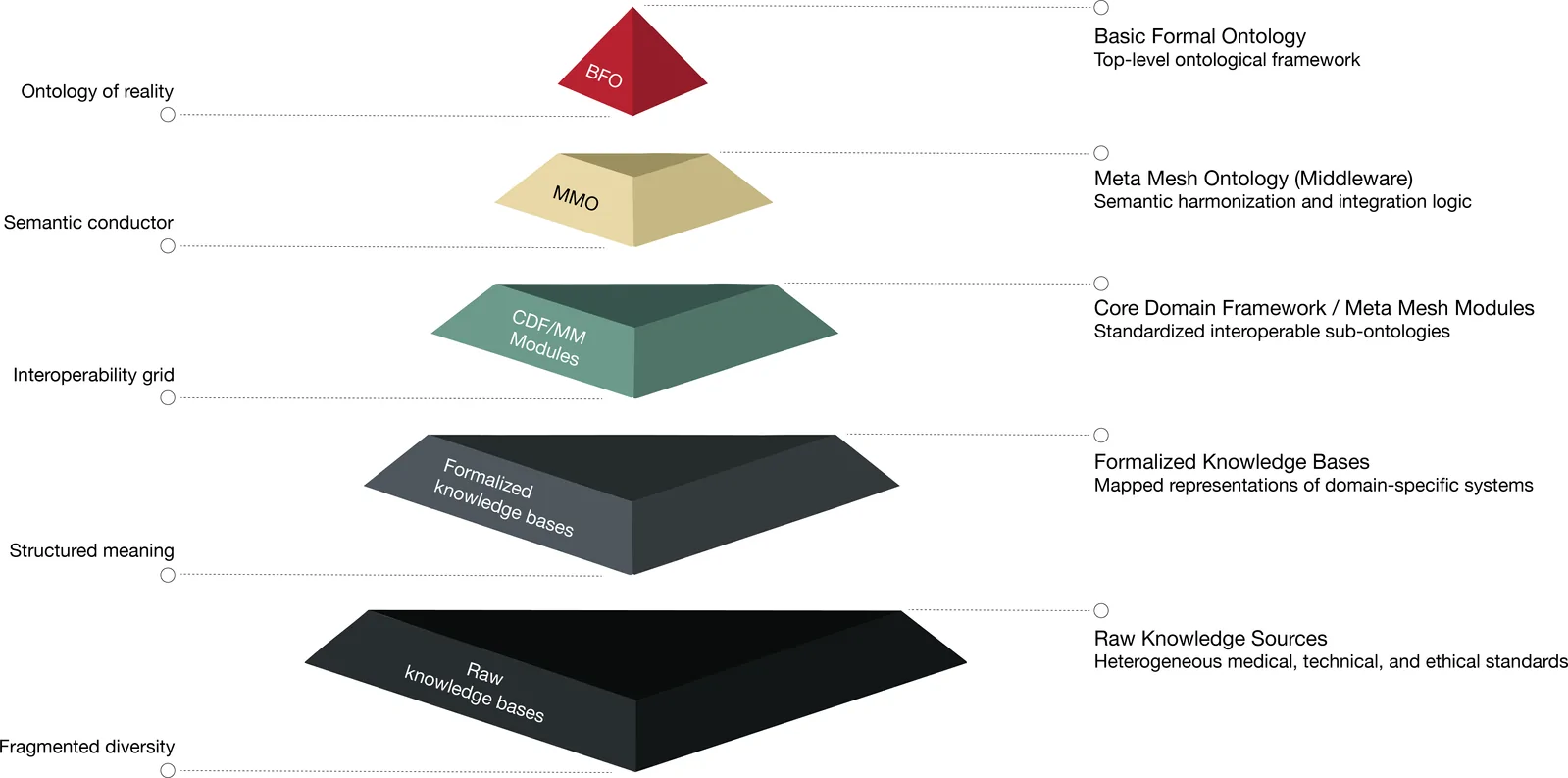
Conceptual layered architecture of the MMO. From bottom to top, heterogeneous raw knowledge sources are represented as formalized knowledge bases, connected through the CDF and MM, mediated by the MMO, and grounded in the BFO. The labels on the left indicate the intended conceptual progression from fragmented diversity and structured meaning to semantic mediation and ontological grounding. BFO: Basic Formal Ontology; MMO: Meta Mesh Ontology; CDF: Core Domain Framework; MM: Meta Mesh Modules.

### Structure and Modular Architecture

Modeling and structuring knowledge is a central challenge in knowledge representation. The goal is to enable a precise and coherent modeling of reality. To achieve this, the MMO uses the BFO as its upper-level ontology [43,44]. The BFO is characterized by a highly axiomatized and formal structure, dividing reality into 2 fundamental categories: entities that persist over time without losing their identity, as well as processes or events that exist within time [45]. This structure is essential for medical knowledge representation, as it allows for the depiction of both states and processes. Building upon this, the MMO establishes a robust foundation, ensuring both structural and semantic consistency. In integrating medical, psychological, and social aspects, the MMO provides a structured framework of concepts and relationships that enables the efficient unification of existing knowledge systems. Based on established metaontological design standards, the MMO was developed according to the following architectural principles to support cross-domain interoperability, modular extensibility, and semantic consistency, which are summarized in Table 2.

**Table 2.** Key architectural principles of the MMO^a^ design.

Key architectural principles	Description
Layered modularity	The MMO^a^ core, the CDF^b^, and the 10 separately versioned domain modules form a layered structure in which each domain module extends the CDF through explicit anchors, so that new domains can be added without modifying the core layers.
Semantic mediation	For each BFO-aligned^c^ upper category, the MMO defines a corresponding subclass that mediates between the upper ontology and domain-specific concepts, providing a shared reference structure through which heterogeneous knowledge representations can be related.
Provenance-preserving alignment	Mappings to external ontologies and terminologies are recorded through explicit predicates (skos:exactMatch, skos:closeMatch, skos:relatedMatch, mmo:isMappedTo, and mmo:isConstantRefinementOf) together with source identifiers and provenance annotations, so that each asserted correspondence remains traceable to its origin.
Validation with version control	Each released version of the MMO core, CDF, and domain modules is checked for OWL^d^ 2 DL profile conformance and logical consistency and is tested through version-controlled competency questions with predefined expected results.

a MMO: Meta Mesh Ontology.

b CDF: Core Domain Framework.

c BFO: Basic Formal Ontology.

d OWL 2 DL: Web Ontology Language 2 description logic.

The modular architecture enables the flexible integration of subontologies and their adaptation to specific application areas. Building on established ontologies and terminologies, this design ensures semantic consistency, compatibility, and seamless interoperability across knowledge systems. To support semantic interoperability across heterogeneous knowledge domains, the MMO uses a CDF [21] as its top-level domain architecture. The CDF defines 8 disjoint but extensible superdomains: knowledge, culture, health, nature, technology, economy, governance, and society, each representing a generalizable and semantically coherent area of human knowledge. These domains serve as anchor points for modular integration and ensure consistent alignment with the BFO. The derivation process followed a multistage synthesis of ontological, epistemological, and classificatory perspectives and is documented in detail in Section S2 in Multimedia Appendix 1. This structured domain scaffold ensures that all domain-specific modules within the MMO can be semantically positioned in a consistent and interoperable way. To illustrate how the CDF integrates into the overall ontology structure, a schematic architectural overview is provided below, as shown in Figure 2. The corresponding class names and unique identifiers are listed in Figure 3.

**Figure 2.**
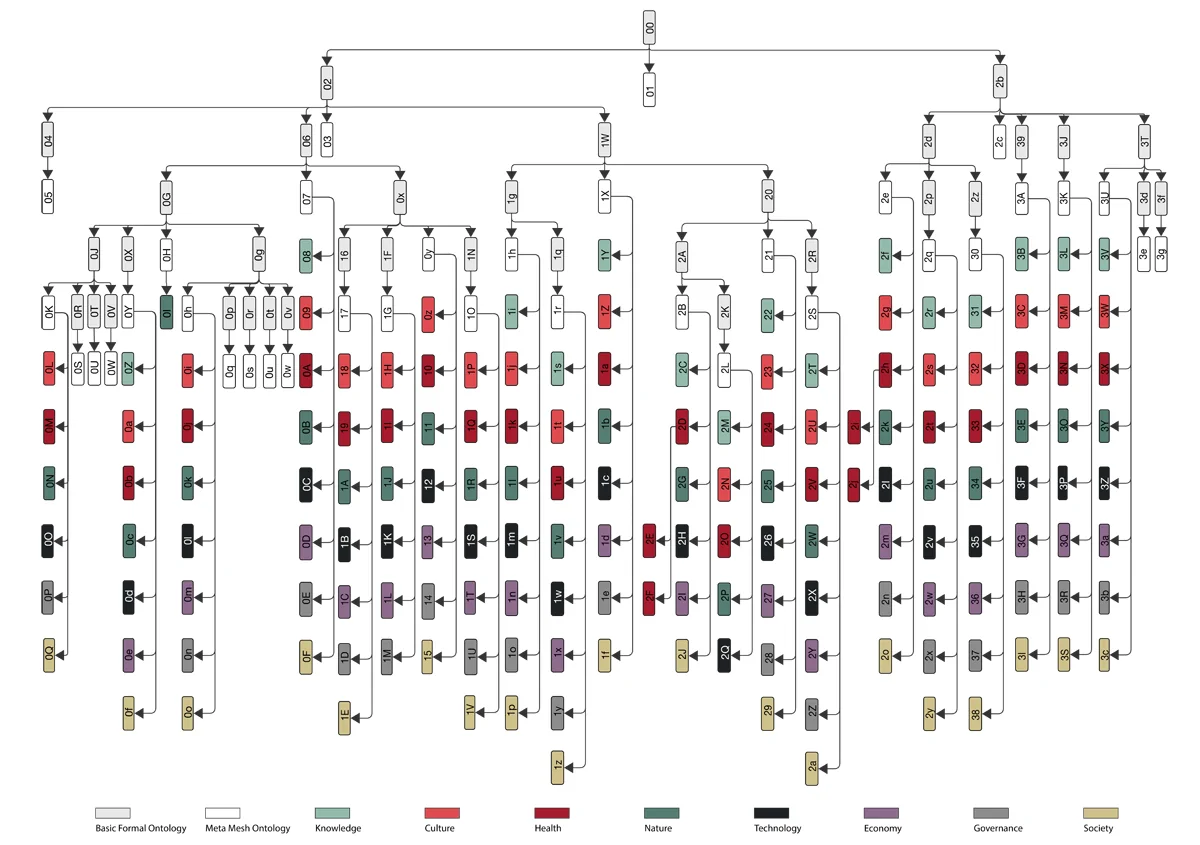
Schematic overview of the Meta Mesh Ontology architecture. The illustration shows the top-level alignment with the Basic Formal Ontology and the integration of the Core Domain Framework classes as intermediary structures that connect domain-specific modules to the foundational layer. The figure illustrates the hierarchical structure and interrelations of the Meta Mesh Ontology and Core Domain Framework classes.

**Figure 3.**
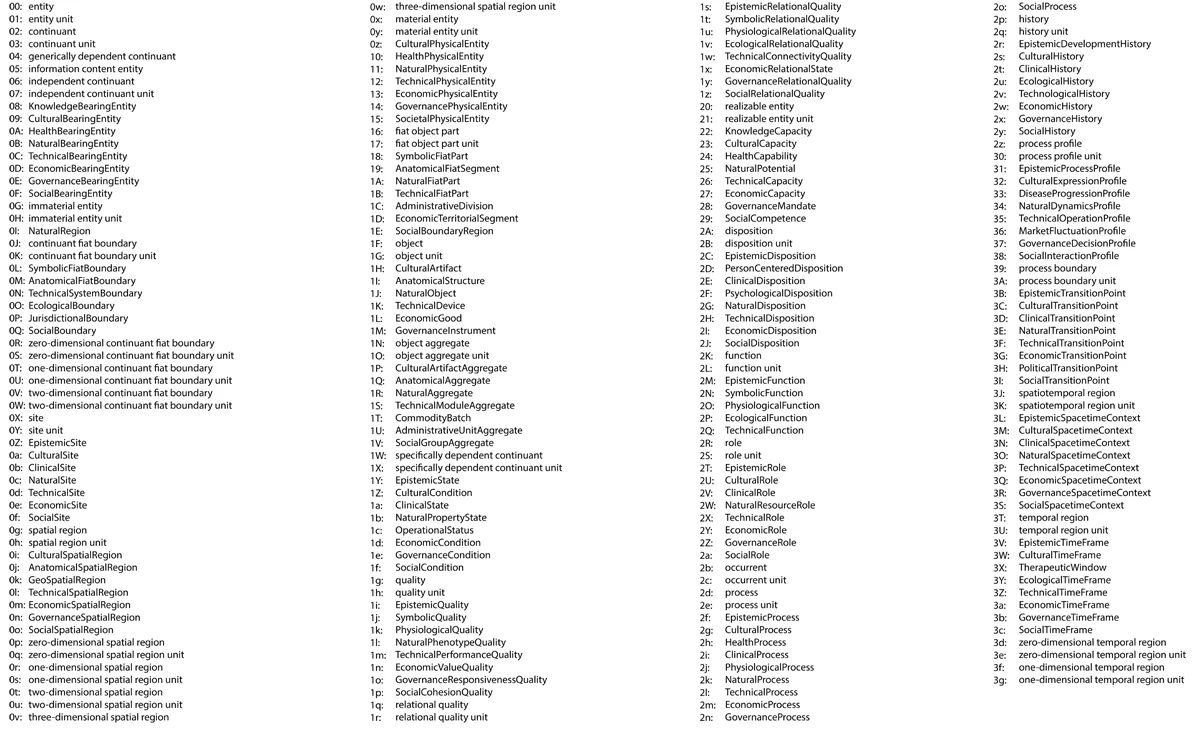
Class-name and identifier key for the Meta Mesh Ontology and Core Domain Framework architecture shown in Figure 2. The figure lists the corresponding class names. Each class is assigned a unique identifier encoded in base62 notation, allowing the abbreviations in Figure 2 to be matched to their respective class names.

### Interoperability and Technical Integration

The MMO supports the harmonization of diverse knowledge domains by providing a set of BFO-compliant classes. For each BFO class, the MMO specifies a corresponding MMO subclass that acts as a semantic mediator and alignment layer. These MMO subclasses serve as supercategories to structure domain-specific concepts and to establish a coherent organization of knowledge domains. In the current evaluation, semantic interoperability was operationalized as machine-readable mappings and their retrieval across the MMO and CDF resources. In this way, the MMO provides explicit mapping structures across the represented knowledge domains. The decision to adopt BFO-compliant categories is intended to enhance both comprehensibility and traceability for users and developers while also facilitating the future integration of new knowledge domains and standards. This results in a framework that can flexibly respond to the rapidly evolving demands of medicine and technology without losing expressiveness or coherence. Ultimately, the ontology is intended to support knowledge management and communication across research, clinical, and technical domains through clearly defined terms and relations. The precise differentiation and modularization of ontology components allow for flexible adaptation to specific use cases and facilitate future developments.

### Synthetic Use Case: Cross-Domain Representation of Mental Health Variables

#### Background

Mental health care frequently faces the challenge of fragmented data landscapes, where critical information is distributed across EHRs, social determinants of health, sensor data (from wearables and smart devices), environmental parameters, and behavioral assessments. This fragmentation makes it difficult to analyze these sources jointly and to preserve their semantic relationships across systems. To illustrate cross-domain representation without using patient-level data, we constructed a synthetic outpatient scenario. This scenario demonstrates how heterogeneous clinical and contextual variables can be represented and semantically linked within the MMO and CDF architecture.

#### Case Description

The case describes a 38-year-old woman presenting to a psychiatric outpatient clinic with sleep disturbances, anhedonia, and fatigue. The scenario includes isolated documentation of previous depressive episodes and somatic complaints. Relevant social data, such as unemployment, single parenting, and housing insecurity, may exist in social service records, while wearable device data would reveal reduced physical activity and fragmented sleep patterns. In conventional clinical workflows, these data remain siloed, inconsistently coded, and lack semantic interoperability. This fragmentation hinders their integration into clinical decision-making processes and constrains the effective use of analytic tools, including but not limited to AI-based methods.

#### Application of the MMO

The MMO was applied in this case to demonstrate the integration of heterogeneous data sources within a digital mental health context. We encoded the outpatient scenario as an RDF graph using identifiers and relations from the MMO core, CDF, and relevant domain modules. The graph brings together clinical, social, environmental, behavioral, sensor-derived, and biomedical variables within a common semantic representation. In total, 50 heterogeneous data points from medical, social, and behavioral domains were incorporated and

- Semantically represented across domains, including medical symptoms, social determinants, trauma history, sensor-based data, biomarkers, molecular data, environmental parameters, behavioral patterns, and care trajectories.
- Mapped to international medical standards, including SNOMED CT [34,35], *ICD-10* [36], LOINC [37,38], UMLS [39,40], SOHO [46], ACESO [47], GO [48], HiTOP [13], and sustainable cities and communities, indicators for city services and quality of life (ISO 37120) [49], using the modular architecture of the MMO and CDF.
- Encoded as a machine-readable, Findable, Accessible, Interoperable, and Reusable (FAIR)-aligned representation with persistent identifiers, source provenance, versioned public access, and documented mappings.

Each variable was represented by an identified RDF resource with type assertions, values or annotations, provenance links, and, where applicable, mappings to module and external entities. For example, the sleep-related variable DP02_Dyssomnia is represented through a SNOMED module class that retains its external identifier and connects it to CDF anchors through explicit mapping and refinement relations; the complete path and query are provided in Section S7 in Multimedia Appendix 1. Competency questions were used to test retrieval through the MMO and CDF layers. Together, these representations link the 50 data points within a common semantic framework. The following use case visualization exemplifies this integration by depicting the full spectrum of data points aligned under the CDF. Detailed information on each of the 50 data points—including domain assignment and semantic encoding, is provided in tabular form in Sections S4 and S5 in Multimedia Appendix 1, with an overview in Figure 4.

All CDF and domain-specific MMO modules used in this study, together with the FAIR-aligned use case representation, are available through the associated versioned repositories [20-23]. Additional documentation, including tabular summaries and semantic encodings, is provided in Sections S4 and S5 in Multimedia Appendix 1.

**Figure 4.**
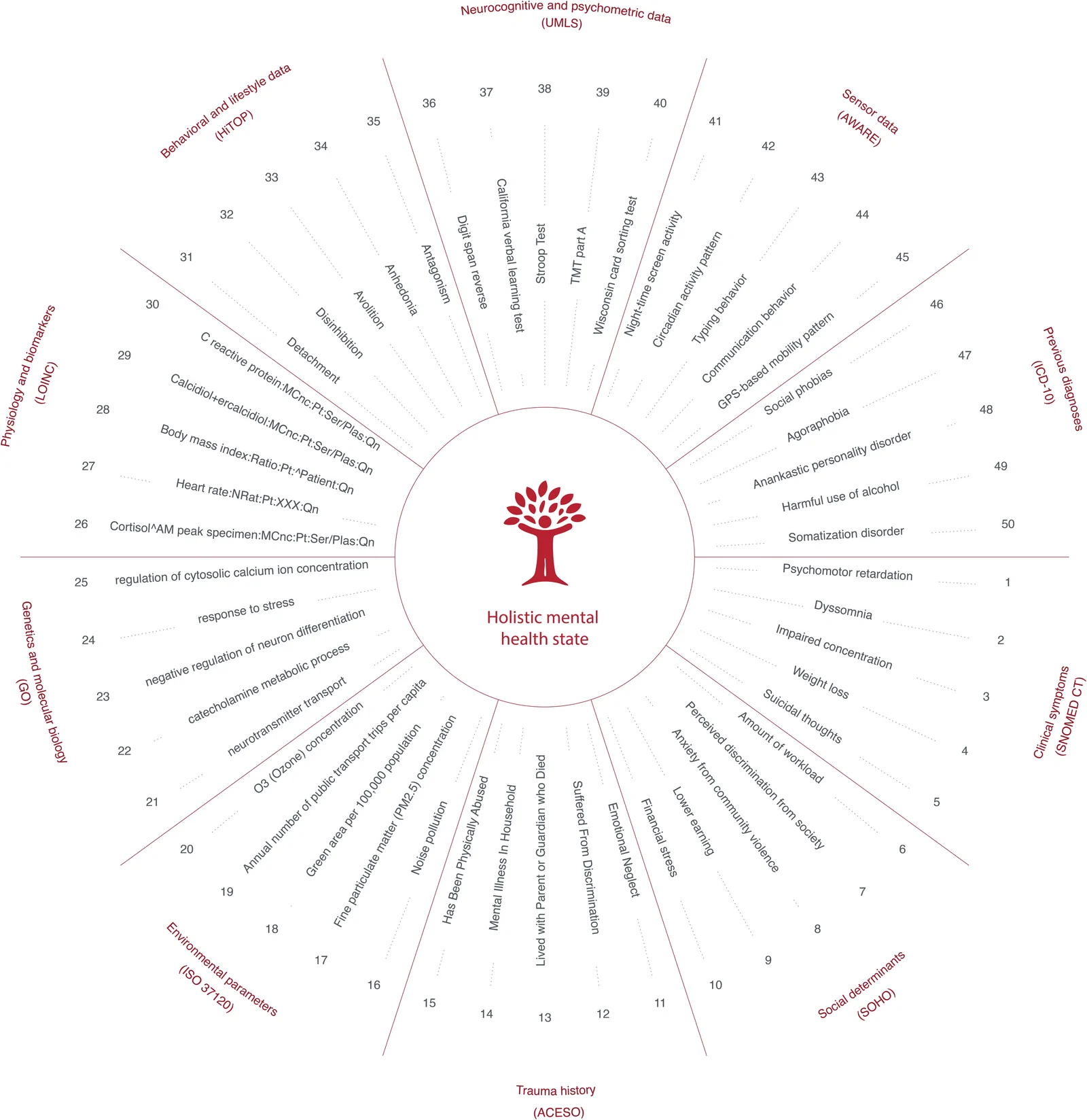
Integrated representation of 50 heterogeneous data points using the Meta Mesh Ontology. The illustration demonstrates how clinical, psychological, environmental, and social information can be unified through domain-level anchoring within the Core Domain Framework. ACESO: Adverse Childhood Experiences Ontology; GO: Gene Ontology; HiTOP: Hierarchical Taxonomy of Psychopathology; *ICD-10: International Classification of Diseases, Tenth Revision*; ISO: International Organization for Standardization; LOINC: Logical Observation Identifiers Names and Codes; MCnc: Mass Concentration; O₃: Ozone; PM2.5: Particulate Matter 2.5; Qn: quantitative (standardized LOINC Property code); SNOMED CT: Systematized Nomenclature of Medicine Clinical Terms; SOHO: Social Determinants of Health Ontology; TMT: Trail Making Test; UMLS: Unified Medical Language System.

### Quantitative Findings

#### Ontology Core Findings

In the combined MMO and CDF analysis, the framework comprised 2238 RDF triples, 208 classes, 28 properties, and 3 individuals. Structural organization was represented by 219 structural relations. We identified 103 semantic alignment relations, including 100 external alignments, across 5 integrated external framework families, which are summarized in Table 3.

**Table 3.** Quantitative summary of MMO and CDF^a^^,^^b^ core metrics^c^.

Metric	Description	Result
RDF^d^ triples	Total number of RDF statements in the ontology.	2238
Classes	Number of ontology classes.	208
Properties	Total number of ontology properties.	28
Individuals	Number of instances.	3
Structural relations	Object properties connecting ontology entities.	219
Semantic internal alignments	Mapping relations linking ontology entities within the MMO and CDF^a^^,^^b^ project space.	3
Semantic external alignments	Mapping relations linking ontology entities to external reference frameworks and terminologies.	100
Integrated frameworks	Number of distinct external framework families represented in the combined MMO and CDF core metrics.	5

a MMO: Meta Mesh Ontology.

b CDF: Core Domain Framework.

c Structural relations denote ontology-internal object-property relations used to organize entities within the MMO and CDF core and should not be interpreted as clinical or outcome-level measures.

d RDF: Resource Description Framework.

These findings indicate that the MMO and CDF core primarily functions as a structurally dense alignment scaffold, with most semantic alignment relations linking outward to external reference frameworks.

#### Ontology Module Findings

The 10-module aggregate added 967 triples, 54 classes, and 20 individuals. Alignment analysis identified 171 semantic alignments (116 external and 55 internal), with 21 module-level ontology integrations, shown in Table 4. Label coverage was complete for all assessed module resources (54/54; weighted coverage 1.0). Module-level complexity varied, with triples ranging from 79 to 183 and total semantic alignments from 9 to 27; the ISO 37120 module had the largest integration burden (183 triples, 27 alignments, and 5 integrated ontologies). Integration counts reflect summed module-level instances across all evaluated modules.

**Table 4.** Module-level ontology and alignment metrics across 10 MMO^a^ modules^b^.

Metric	Description	Result
RDF^c^ triples	Total number of RDF statements in the ontology.	967
Classes	Number of ontology classes.	54
Individuals	Number of instances.	20
Semantic internal alignments	Mapping relations linking module entities to MMO and CDF^a^^,^^d^ internal project space entities.	55
Semantic external alignments	Mapping relations linking module entities to external reference frameworks and terminologies.	116
Module-level ontology integration instances	Summed count of module-level ontology integration instances across all evaluated modules.	21

a MMO: Meta Mesh Ontology.

b The value “21 integrations” represents the sum of module-level ontology integration instances across 10 evaluated modules and does not indicate 21 unique external frameworks. The 54/54 coverage value refers to complete label coverage across assessed module resources and should be interpreted as an ontology-side documentation/readiness signal rather than direct evidence of empirical dataset interoperability.

c RDF: Resource Description Framework.

d CDF: Core Domain Framework.

The range of module-level counts reflects the deliberately differentiated scope and modeled content of the 10 exemplar modules.

### Logical and Functional Validation

The MMO core, CDF, and all 10 domain modules conformed to the OWL 2 DL profile, and HermiT reasoning through ROBOT version 1.9.6 detected no logical inconsistency. Observed results matched the expected results for all 11 competency questions. The tests retrieved all 50 data points, traced each data point through its module class to at least 1 CDF anchor, confirmed source provenance for the represented module classes, and produced the selected expected OWL entailment. All 4 negative controls were detected as expected. Complete test specifications, expected and observed results, queries, validation reports, and reproduction instructions are summarized in Sections S6 and S7 in Multimedia Appendix 1 and provided in full in the versioned evaluation release [23].

## Discussion

### Principal Findings

We developed and released the MMO and CDF architecture and examined its structure, asserted mappings, logical consistency, and answers to specified competency questions based on a mental health scenario. In that scenario, the graph represented clinical, psychosocial, environmental, and biomedical variables while retaining their source identifiers.

### Strengths and Limitations

#### Strengths

The MMO has several strengths as a modular ontology architecture. Beyond its theoretical foundation, the MMO provides an implemented and versioned framework for linking heterogeneous knowledge representations through explicit mappings. The quantitative profile further indicates a 2-layer integration pattern consisting of a stable MMO and CDF core and a heterogeneous domain-module layer with substantial external alignment density. Taken together, these findings support an interpretation in which the core provides the semantic scaffold, while the module layer enables domain-specific linkage to external clinical, psychosocial, and environmental reference systems. This structure supports modular extension through separately versioned domain modules while preserving the distinction between the core and module layers. By representing medical, technological, environmental, and socioeconomic concepts through standardized domain modules, including concepts related to smart devices, sensors, EHRs, and patient-reported outcomes, the MMO provides a multidimensional semantic structure. This structured networking of knowledge representations brings biological, psychological, social, and environmental concepts into a common ontology architecture. Another key advantage of the MMO lies in its explicit mappings among source terminologies, which retain source identifiers and provenance across the domain modules. The module architecture includes explicit references and mappings to international standards and medical terminologies, represented in this study, including *ICD-10*, SNOMED CT, UMLS, LOINC, and ISO 37120. Within the current evaluation scope, the reported metrics and functional tests characterize ontology structure, alignment density, cross-layer retrieval, and selected entailment behavior. The MMO’s BFO grounding aligns it with other realist, BFO-based approaches to mental health knowledge representation, while its modular mediating layer addresses a gap that these approaches do not cover. The Mental Functioning Ontology and its Mental Disease module (MFO-MD) [50,51] similarly ground mental-health concepts in BFO and reuse external identifiers, including from the Disease Ontology, but their scope is limited to mental functioning and disease; unlike the MMO, their published documentation does not describe a mechanism for anchoring modules from unrelated domains, such as social determinants or environmental indicators, within a shared mediating layer. The OBO Foundry [52] pursues BFO-based interoperability across a coordinated family of independently developed ontologies, but does so through shared design principles and a registry rather than through a single architecture with a versioned anchor layer and explicit, provenance-bearing mapping predicates between member ontologies, both of which the MMO provides. BioPortal [28], which indexes and serves ontologies such as SNOMED CT, *ICD-10*, and LOINC, functions as a repository and access platform rather than as a mediating ontology in its own right. The MMO’s contribution within this landscape is therefore a comparatively lightweight but concrete mediating layer that already spans domains with no prior mental health orientation, such as environmental and urban indicators, under one-versioned and evaluable structure.

#### Limitations

A key limitation of the current evaluation is that it primarily captures structural and semantic integration characteristics of the ontology rather than downstream clinical-performance outcomes. The current metrics characterize ontology structure, alignment density, and ontology-side documentation readiness, but they do not by themselves demonstrate downstream clinical utility, true dataset interoperability, or patient-level performance effects. In addition, the readiness indicators observed in the metrics analysis were mixed to moderate, and reasoning and competency-question tests cover only the specified test cases. The use case evaluates specified mapping, retrieval, integrity, and entailment behavior but does not assess mapping completeness, independent mapping validity, or application to governed clinical data. Query performance under production workloads and downstream clinical or machine-learning outcomes were not evaluated. Beyond these methodological constraints, several considerations would apply to future operational implementation. Implementing ontology-mediated interoperability in operational health care settings may require investment and adjustments to existing technical and organizational structures. Such implementation may also require training for professionals working with the relevant systems. Any future integration and networking of personal data would require appropriate data protection and security measures. Ethical and legal requirements, including applicable data-protection standards, would also need to be addressed. Technical complexity may present another implementation challenge. Many existing systems are proprietary and not designed for seamless interoperability. Harmonizing and standardizing these systems requires not only technological adjustments but also institutional and regulatory agreements, which are not always easy to implement. Furthermore, the MMO requires continuous maintenance and regular updates to ensure its long-term usability and acceptance. Future implementations in non-WEIRD (Western educated, industrialized, rich, and democratic) contexts involve additional challenges, as limited resources, differing regulatory frameworks, and varying digital infrastructures can hinder large-scale adoption. Therefore, implementation strategies should consider local capacities, sociotechnical conditions, and culturally adapted governance models to ensure equitable accessibility and long-term sustainability.

### Implications and Future Evaluation

Beyond these structural findings, the approach suggests several directions for future research in psychiatric knowledge representation and ontology-informed analysis. Future studies could evaluate whether MMO-based representations facilitate the joint analysis of heterogeneous information sources in psychiatric research and clinical workflows. By integrating semantic concepts, various psychiatric classification systems, taxonomies, and frameworks, such as *DSM*, *International Classification of Diseases* (*ICD)*, HiTOP, and RDoC, can be harmonized and linked to digital systems. Future research could examine the use of these mappings for longitudinal representation and analysis. The module architecture can also represent concepts related to smart devices, wearables, and other technology-supported sources. Wearable technologies and sensors continuously provide psychophysiological data such as heart rate variability, sleep patterns, and activity levels. Implementation studies could assess whether such observations can be represented consistently alongside clinical and contextual information. Separately developed decision-support or machine-learning systems could evaluate MMO-structured representations as one form of semantic input. Future studies could examine ontology-mediated representations in telemedicine and digital therapy applications. Such studies should separately evaluate information exchange, workflow integration, and clinical utility. Additionally, the ontology contributes to designing health-promoting environments in urban areas. By linking smart city data with psychiatric care models, environmental factors that influence mental well-being can be analyzed to develop evidence-based infrastructural interventions.

Taken together, these considerations suggest that the MMO represents a strategic opportunity to transform psychiatric diagnostics and therapy. Its potential lies not only in the semantic interoperability and standardization of clinical and technological data but also in creating an infrastructure that enables the sustainable and evidence-based advancement of psychiatry. A key aspect of the MMO is the establishment of a methodological foundation that allows the integration of heterogeneous data sources for both scientific and clinical purposes. This involves not only the harmonization of existing classification systems such as *DSM-5*, *ICD*, HiTOP, and RDoC but also the structured incorporation of new insights gained from AI-driven analyses into diagnostic and therapeutic processes. In summary, the MMO can be regarded as a forward-thinking concept that may support data-driven, interdisciplinary, and ethically grounded psychiatry; however, the current findings are ontology-structural and do not constitute evaluated operational reasoning performance, downstream model performance, or patient-level clinical effectiveness.

## Supplementary material

10.2196/96311 Multimedia Appendix 1 Core Domain Framework (CDF) derivation, Meta Mesh Ontology (MMO), and CDF class definitions, synthetic use-case mappings, competency-question validation, negative controls, and a worked mapping example.

## Abbreviations

ACESO: adverse childhood experiences ontology
BFO: basic formal ontology
CDF: Core Domain Framework
*DSM-5*: *Diagnostic and Statistical Manual of Mental Disorders, Fifth Edition*
EHRs: electronic health records
FAIR: Findable, Accessible, Interoperable, and Reusable
GO: gene ontology
HiTOP: Hierarchical Taxonomy of Psychopathology
*ICD-10*: *International Classification of Diseases, Version 10*
*ICD-11*: *International Classification of Diseases, 11th Revision*
ISO: International Organization for Standardization
LOINC: Logical Observation Identifier Names and Codes
MFO-MD: Mental Functioning Ontology-Mental Disease module
MMO: Meta Mesh Ontology
OBO: open biological and biomedical ontologies
OLS: Ontology Lookup Service
OWL: web ontology language
OWL 2 DL: Web Ontology Language 2 Description Logic
OWL-RL: Web Ontology Language 2 Rule Language
RDF: Resource Description Framework
RDoC: Research Domain Criteria
SKOS: Simple Knowledge Organization System
SNOMED CT: Systematized Nomenclature of Medicine Clinical Terms
SOHO: Social Determinants of Health Ontology
SPAQRL: SPARQL Protocol and RDF Query Language
SWRL: Semantic Web Rule Language
UMLS: Unified Medical Language System
WEIRD: Western educated, industrialized, rich, and democratic
